# Timing of lung transplant evaluation: considerations, barriers and alternatives

**DOI:** 10.1097/MCP.0000000000001176

**Published:** 2025-04-25

**Authors:** Eline A. van der Ploeg, Tjerk H. Hylkema, C. Tji Gan

**Affiliations:** Department of Respiratory Diseases, Tuberculosis and Lung Transplantation, University of Groningen, University Medical Center, Groningen, The Netherlands

**Keywords:** evaluation, lung transplantation, respiratory disease

## Abstract

**Purpose of review:**

Over the past decade, increased knowledge has contributed to improved medical and technical treatments across the spectrum of respiratory diseases. As a result, timing for transplant evaluation might be more challenging. In this review, the focus is on timing of lung transplant evaluation of patients from the main respiratory diseases referred. Disease-specific predictors of survival in relation to timing of transplant evaluation and alternative treatments will be reviewed.

**Recent findings:**

Treatment options have evolved for respiratory diseases like chronic obstructive pulmonary disease, pulmonary fibrosis, cystic fibrosis and pulmonary arterial hypertension. These treatments have led to improved quality of life, exercise tolerance, lung function and outcome. However, the effect of these alternative treatments on transplant candidacy and knowledge on timing of lung transplant evaluation are lacking.

**Summary:**

This article reviews the current best evidence to guide clinicians regarding the optimum timing for transplant referral and highlights considerations to optimize transplant candidacy and outcomes.

## INTRODUCTION

The last decades’ knowledge of specific respiratory diseases has moved the field forward. Excellent research with a focus on pathobiology, biomarkers and genetics has led to better understanding of respiratory diseases and opened doors to development of specific medical treatments [1–4]. In addition, with developments in medical technologies, the future still holds many potential challenges yet to be solved. As a consequence, new treatments might stabilize the disease and result in improved quality of life and survival. Despite significant advances in disease-specific therapies, lung transplantation remains the only therapeutic option for end-stage respiratory failure. However, because of improved quality of life and survival, the need for a lung transplantation (LTx) might be less urgent, and the timing for evaluation and transplantation remains challenging.

In this review, the focus is on timing of lung transplant evaluation of patients from the main respiratory diseases referred for LTx chronic obstructive pulmonary disease (COPD), pulmonary fibrosis, cystic fibrosis and pulmonary arterial hypertension (PAH). We will evaluate disease-specific predictors of survival in relation to timing of transplant evaluation, potential challenges and alternative treatments which might be considered. 

**Box 1:**
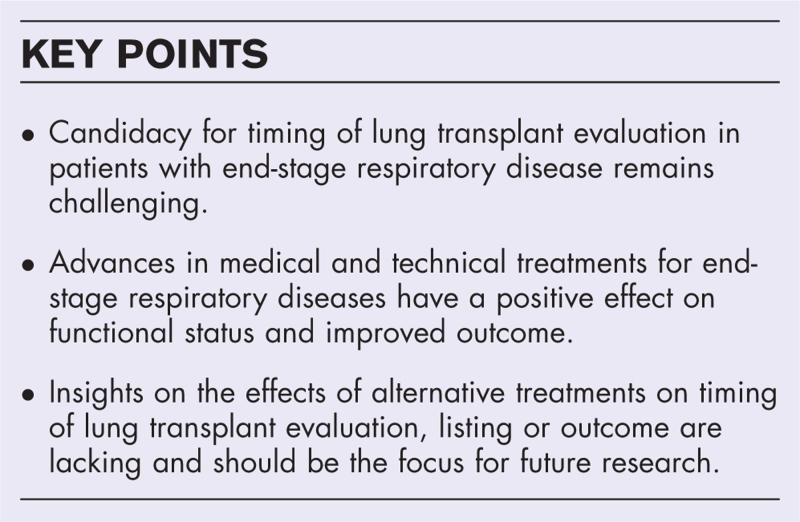
no caption available

## CHRONIC OBSTRUCTIVE PULMONARY DISEASE

Timing of transplant evaluation and listing remains a challenge in COPD patients. Patients with poor quality of life due to further clinical deterioration despite optimization of specific treatment, and those with increased mortality risk should be considered for transplant evaluation and listing. The Global Initiative for Chronic Obstructive Lung Disease (GOLD) support the BODE index as most accurate predictive model [1]. This 10-point model includes multiple parameters covering variables showing physiological reserve (BMI and airflow obstruction), exercise limitation (6-min walk distance) and dyspnoea score (Medical Research Counsil Dyspnoea scale) [5]. More simple models or even single predictors of mortality have been less robust than a multivariable predictive model. The BODE index is embraced by the International Society of Heart and Lung Transplantation (ISHLT), and in the most recent guidelines, it plays an important role [6]. A patient with a BODE score 5–6 should be considered for transplant evaluation. Compared to the initial consensus guidelines [7], additional factors, which are associated with increased mortality in COPD are added. These are forced expiratory volume in one second (FEV1) 20–25%, frequent acute exacerbations, an increase in BODE score greater than 1 point in the last year, signs of pulmonary hypertension on computed tomography (CT) scan shown by a pulmonary artery to aorta diameter greater than 1. Not only poor quality of life and further clinical deterioration despite optimization of medical treatment but also maximization of COPD-specific treatment, such as pulmonary rehabilitation, oxygen therapy or even noninvasive ventilation when indicated, warrants referral for lung transplant evaluation. Although the accuracy of BODE index has been debated and has shown to be inconsistent with respect to prediction of mortality in large COPD cohorts [8], a BODE score 5–6 may guide the general pulmonologist when to refer the patient for LTx evaluation.

General considerations that pose the risk and benefit of a LTx should be taken into account when referred. Age, body weight, functional status, comorbidity, that is, cardiovascular disease, renal insufficiency, coronary artery disease, gastrointestinal dysfunction, psychological risk factors, infectious disease should all be investigated thoroughly. In COPD patients, some of these general considerations might be more worrisome. With increasing age and thus potentially more comorbidity of the potential lung transplant candidates, some of the general considerations should be placed in a broader perspective. The prevalence of cardiovascular disease in COPD might be as high as 70% [1]. Management and treatment at the time of transplant evaluation, and listing is a prerequisite. There is significant increased cardiovascular disease risk post LTx in general [9]. Due to high prevalence of cardiovascular disease in COPD as a consequence of systemic inflammation and smoking, the risk of development or progression of cardiovascular disease post LTx might, therefore, be higher. Furthermore, osteoporosis in COPD is common despite treatment [10]. In addition to the side effects of steroids, insufficient diet and sedentary lifestyle might negatively affect bone density in COPD. Unfortunately, there are no specific data on cardiovascular disease or osteoporosis in COPD post LTx to draw any firm conclusion to be a ‘barrier’ for evaluation or listing. Although LTx improves quality of life in COPD, cardiovascular disease and osteoporosis might have an effect on quality-of-life post LTx. Tobacco exposure in COPD is not only associated with the development of lung cancer but also increases the risk for other types of cancer such as colon and oropharyngeal cancers [11]. Obviously, this may impair prognosis, but this increased risk itself is not a definite barrier for LTx.

General and or specific considerations in COPD patients may form potential barriers to proceed to LTx. However, there might also be nonpatient-related barriers in the transplant trajectory. Globally, there is a trend where patients with pulmonary fibrosis and older age are prioritized for transplantation [12^▪^]. There are different allocation systems, but generally, they are meant to prioritize the most ill patients to reduce waitlist mortality and improve survival [12^▪^]. Modifications to allocation systems aimed not only at long-term outcome but also quality of life is warranted. With the trend of transplantation of older fibrotic male patients, the waiting list time for COPD patients might be prolonged with consequences for age, comorbidities, increased transplant risk and questionable rehabilitation outcomes post LTx, which might affect long-term outcome.

To optimize transplant candidacy, additional treatment options should be considered in COPD and might eventually be regarded as an alternative for LTx. Pharmacotherapy and management of treatable traits are aimed to control symptoms, reduce the rate of exacerbation and prevention of disease progression [13]. A more advanced treatment in COPD with hypercapnia (pCO_2_ > 6.0 kPa) is noninvasive positive pressure ventilation (NPPV). It improves quality of life, reduce exacerbation frequency and has a survival benefit in COPD patients with more severe hypercapnia (pCO_2_ > 7 kPa) [14,15^▪^]. In addition, COPD patients with the most emphysematous phenotype and static hyperinflation might benefit from either surgical or endoscopic lung volume reduction (ELVR). Candidate selection is important. Where these techniques might be considered in patients with a residual volume at least 75% and a ratio of residual volume and total lung capacity the ratio of residual volume and total lung capacity at least 50%. A chest CT is crucial to evaluate the severity and distribution of emphysema [16]. The surgical technique has shown to improve exercise capacity and quality of life [17]. For ELVR, endobronchial valve placement is an accepted treatment in highly selected candidates [18,19]. ELVR has shown to reduce hyperinflation and improved exercise capacity and quality of life. LVR should be considered as an alternative to LTx, but due to the loss of clinical benefit after years [20], alternative treatments as described might also be considered as bridging strategies to LTx.

## PULMONARY FIBROSIS

Pulmonary fibrosis is one of the leading indications for LTx worldwide. Pulmonary fibrosis is a result of, for example, idiopathic interstitial pneumonias (IIP), connective tissue disease-related interstitial lung disease (CTD-ILD), hypersensitivity pneumonitis or sarcoidosis. Idiopathic pulmonary fibrosis (IPF) is the archetypical progressive fibrotic ILD, and patients with IPF are commonly referred for LTx, as mortality rates are high. In end-stage pulmonary fibrosis, patients have low diffusion capacities and low forced vital capacities (FVC). Moreover, around 40% of the end-stage IPF patients have developed pulmonary hypertension [21]. Without treatment, pulmonary fibrosis patients have a survival rate of approximately 3–4 years [22]. Therefore, timely referral to a transplantation centre is highly recommended due to their poor prognosis, high mortality on the waiting list and the unpredictable disease course. In several previous consensus documents of expert groups, timely referral is recommended for IPF and non-IPF patients [6,23]. Disease progression, impaired lung function FVC less than 80% or DLCO less than 40%, relative decline of FVC or DLCO with or without worsening of symptoms and radiology, and administration of supplemental oxygen are all hallmarks indicating it is time to evaluate for LTx.

However, earlier referral provides opportunities to address BMI issues, treat/prevent osteoporosis, avoid deconditioning and enable forming a relationship between transplant team personnel and patients, all aiding in improving outcomes [23]. In the past years, new developments in treatment for pulmonary fibrosis are observed. Two landmark trials published in 2014, showed that antifibrotic therapies Nintedanib and Pirfenidon were successful for IPF patients to stabilize the fibrotic changes [24,25]. This resulted in a slower decline of FVC. Other studies have shown that antifibrotic therapy also prolongs survival among IPF patients [24,26,27]. However, the impact of antifibrotic therapy on timing of referral to transplantation centres is not known yet. One could argue that referral will be postponed due to the stabilization of the disease and therefore a lower need for transplantation. Postponing referral will lead to older patients undergoing transplantation. However, outcome of LTx do not differ between patients with IPF younger than 65 years and younger than 70 years [28], and it remains debated what the age cut-off is to perform LTx. Whether patients are receiving antifibrotic therapy or not, referring patients early in the disease process is still warranted.

## CYSTIC FIBROSIS

Over the years, the life expectancy of cystic fibrosis patients improved because of early diagnosis, advances in antimicrobial treatment, nutritional improvement and mucus clearance [3]. Since the advent of cystic fibrosis transmembrane conductance regulator (CFTR) modulators, the quality of life of cystic fibrosis patients has improved dramatically. CFTR modulators improve the function of this defective protein in patients who have at least one F508del mutation of the gene. CFTR modulators improve pulmonary function and decreases the number of exacerbations [29]. However, around 10% of the patients do not benefit from these modulators because of other genotypes affected or side effects [30]. Timing of evaluation for LTx in adult cystic fibrosis patients is mainly based on FEV1. Decreased FEV1 is related to mortality in cystic fibrosis [31,32]. There are currently no risk models to guide patient referral for Ltx in cystic fibrosis. The ISHLT consensus guideline on selection of patients for LTx does recommend referral for patients who have an FEV1 of less than 30% despite optimal medical treatment, and if appropriate, had a trial of CFTR modulators. Also, patients with FEV1less than 40% and a 6 min walking test below 400 m, or PaCO_2_ greater than 50 mmHg (6.65 kPa), or hypoxemia at rest or with exertion can be referred. Other criteria included are pulmonary hypertension (estimated pulmonary artery systolic pressure of 50 mmHg at echocardiogram or right ventricular dysfunction), aggravating nutritional status despite suppletion, two or more hospitalizations for exacerbations requiring intravenous antibiotics, haemoptysis needing embolization and pneumothorax. Patients with a FEV1 of less than 50% and are deteriorating fast should be referred for LTx evaluation. The need for NPPV during an exacerbation is also an indication for referral [6].

Evaluation of cystic fibrosis patients for LTx should include cultures of bacterial and fungal pathogens carried by the patient. Especially *pseudomonas aeruginosa*, *Burkholderia cepacia* complex, *Scedosporium/Lemenospora* and nontuberculous mycobacteria carried by cystic fibrosis patients such as *Mycobacterium avium* and *Mycobacterium abcessus* are associated with lung function decline and with increased risk of death before LTx [33,34]. These microorganisms can be a barrier to candidacy/listing for LTx in nonspecialist centres. Low BMI is associated with lung function decline and mortality in cystic fibrosis [34], and even though, it is not associated with posttransplant mortality [35], it is advised to preferably optimize BMI to more than 17 kg/m^2^ pretransplantation. Liver dysfunction and cholestasis is common in cystic fibrosis patients and should be carefully evaluated before LTx to avoid complications.

Due to the introduction of CFTR modulators and yet unknown long-term effects, it is possible that new criteria need to be established for referral in the cystic fibrosis patient population in the near future.

## PULMONARY ARTERIAL HYPERTENSION

Pulmonary arterial hypertension (PAH) is a condition marked by remodelling of the pulmonary arterial system, which ultimately leads to right ventricular failure and death [36]. Treatment for PAH is currently mainly targeted at vasodilatation and decrease of pulmonary vascular resistance. No treatments exist that can reverse or halt vascular remodelling. PAH patients with vasoreactivity at right heart catheterization can be treated with calcium channel blockers (CCB). The prognosis in long-term responders to CCBs is much better compared with nonresponders. However, only 10% of PAH patients are vasoreactive [37].

The advice in case of nonvasoreactivity is to start on dual therapy with an endothelin receptor antagonist together with a phosphodiesterase-5 inhibitor. Initial triple therapy with infused prostacyclin analogue should be considered in high-risk patients [38]. Sotatercept, targeting dysregulation of bone morphogenetic protein receptor type 2 signalling pathway, is upcoming and shows promising results in PAH increasing 6-min walk test and lowering pulmonary vascular resistance [39^▪^].

Early referral for patients with PAH is warranted because of the risk for sudden and fast deterioration due to acute right ventricular failure. Waitlist mortality in countries using the LAS is high, due to the ineffective reflection of severity of the condition for this group of patients [40]. A high urgency allocation for patients with PAH improved waitlist mortality in PAH [41]. Waitlist mortality and posttransplantation survival was also improved by the use of preoperative and peri-operative extracorporeal membrane oxygenation (ECMO). Veno-arterial ECMO should thus be considered as a bridge to transplant in patients who need right ventricular support [42].

In the ISHLT 2021 guideline, several clinical criteria, as well as the use of a risk model are advised. The Registry to Evaluate Early and Longterm Pulmonary Artery Hypertension disease management (REVEAL) risk model is a validated tool to evaluate the 1-year survival in recently diagnosed PAH patients and is used to guide medical treatment. Incorporating multiple criteria, such as SBP, heart rate and hospitalizations, the model has good longitudinal prediction value for survival and thus can also be used to assess disease progression in PAH despite optimal treatment [43].

Current criteria for referral include all potentially eligible patients for LTx in case of treatment failure. So, patients who are on appropriate therapy but fail to achieve symptom control with progressive breathlessness or show signs of right ventricular failure, indicated by, for example, oedema and ascites or progressive hypoxemia, should be evaluated for LTx. The European Society of Cardiology/European Respiratory Society as well as the ISHLT also state patients with a REVEAL risk score of 7 (ISHLT of 8) on appropriate PAH medication should be referred [6,38]. These are patients that are still in the intermediate–high to high-risk groups according to REVEAL for survival within 1 year despite treatment (with subsequent survival rates of 70–94%, <70%). Other reasons for referral include progressive disease, significant right ventricular dysfunction, signs of secondary liver or kidney dysfunction due to PAH, other complications such as haemoptysis, or recent hospitalization for PAH. Another main reason is need for intravenous or subcutaneous prostacyclin therapy.

## CONCLUSION

In conclusion, LTx remains a definite treatment option in patients with an end-stage respiratory disease. Due to development of medical and technological treatments, the perfect timing for evaluation is even more challenging. In the near future, the focus should be on the long-term effects of these medical and technological treatments in the light of LTx candidacy timing. These evolving new insights might give more guidance to the general pulmonologist for the timing of transplant referral.

## Acknowledgements

*None.*

### Financial support and sponsorship

*None.*

### Conflicts of interest

*There are no conflicts of interest.*

## REFERENCES AND RECOMMENDED READING

Papers of particular interest, published within the annual period of review, have been highlighted as:

▪ of special interest
▪▪ of outstanding interest
